# Sinonasal malignancies: histopathological entities, regional involvement and long-term outcome

**DOI:** 10.1186/s40463-023-00627-8

**Published:** 2023-04-28

**Authors:** Lukas Anschuetz, Ralph Hohenberger, Christoph Kaecker, Olgun Elicin, Roland Giger, Marco Caversaccio

**Affiliations:** 1 Department of Otorhinolaryngology, Head and Neck Surgery, Inselspital, Bern University Hospital, University of Bern, Freiburgstrasse 10, 3010 Bern, Switzerland; 2 Department of Otorhinolaryngology, Head and Neck Surgery, Heidelberg University Hospital, Heidelberg, Germany; 3 Department of Radiation Oncology, Inselspital, Bern University Hospital, University of Bern, Bern, Switzerland

**Keywords:** Nasal cancer, Mucosal melanoma, Squamous cell carcinoma, Regional recurrence, Rare cancer

## Abstract

**Background:**

To assess a large patient cohort with sinonasal malignancies focusing on regional involvement, recurrence and oncological outcome.

**Methods:**

Patients (n = 144) with malignant tumors of the nasal cavity and paranasal sinuses were treated at our tertiary referral center between 2008 and 2019. A chart review on patient and tumor characteristics, treatment and long-term outcome was performed.

**Results:**

Most frequent histological types were squamous cell carcinoma (SCC) (n = 74), adenocarcinoma (n = 24) and mucosal melanoma (n = 18). Primary therapy was surgery in 66% of patients (n = 95) of which 65.8% (n = 66) received adjuvant radiotherapy. Twenty patients (13.8%) were initially staged as cN + and in seven cases, pN + status was histopathologically confirmed. Fifty-six of 130 patients (43.1%) had a relapse after curative intended therapy, including nine loco-regional (6.9%) and seven isolated regional recurrences (5.4%). Twelve of these 16 patients with (loco-)regional recurrence had SCC. Adenoid cystic carcinoma (87.5%) and SCC (65.3%) showed the best long-term overall survival.

**Conclusions:**

Regional involvement and regional recurrence are scarce. Because of rarity and heterogeneity, evidence on therapeutic management is sparse resulting in the lack of clinical guidelines.

**Graphical Abstract:**

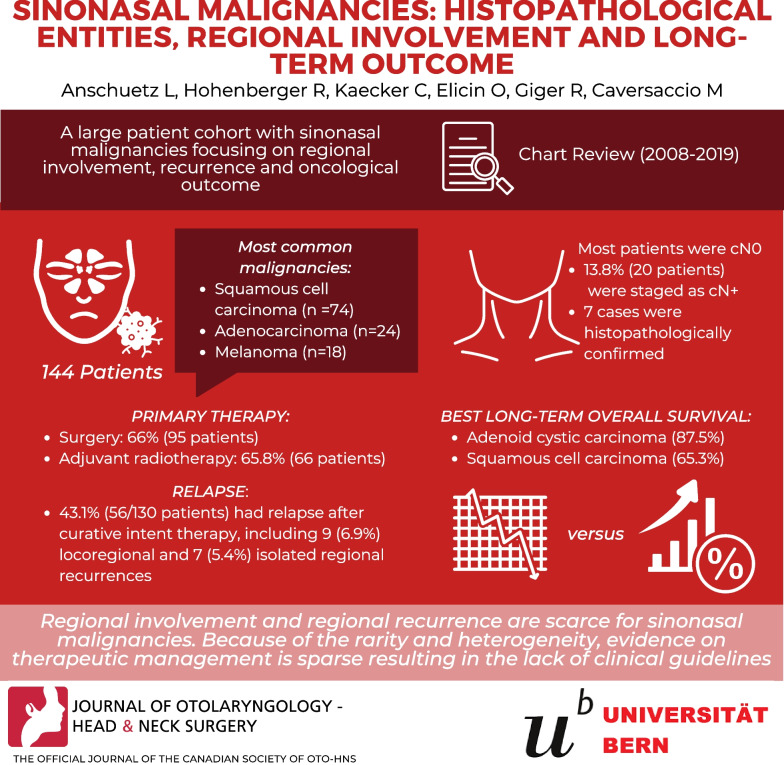

## Background

Sinonasal malignancies are a group of rare tumors with a considerable histopathological heterogeneity. The most common entities are squamous cell carcinoma (SCC), adenocarcinoma and mucosal melanoma (MM) [1, 2]. The prognosis depends on initial tumor stage and histological subtype. Sinonasal MM in particular have a poor prognosis with 5-year overall survival (OS) rates not exceeding 42% [3–5]. Other entities have a better 5-year OS with up to 92% for SCC [6]. These tumors belong to the domain of rare cancer according to the RARECARE classification and ICD10 [7, 8]. There is a lack of evidence from prospectively collected data for treatment, especially for histological subtypes other than SCC and for management of the lymph nodes [9]. Data, preferably from large patient cohorts could therefore be interesting to refine management strategies and to adequately counsel patients.

For treatment of the primary tumor in non-metastatic stage, surgery is the mainstay and provides good local control for histological subtypes other than MM. Over the last two decades, endoscopic approaches for sinonasal malignancies have proven equal to an open approach in terms of practicality and efficiency in selected patients and are associated with lower complication rates [1, 10, 11]. Adjuvant (chemo)radiotherapy ((C)RT) is reported to improve survival rates, while positive surgical margins or primary non-surgical treatment are factors for worse outcomes [2, 12, 13].

Initial regional involvement is uncommon and no conclusive evidence for the management of the neck lymph nodes is available. Most patients are staged cN0 and even in many patients with suspected regional involvement (cN +), consecutive histopathological findings were often found negative [2, 6, 14, 15]. In some cohorts of nasal SCC, regional treatment was reserved for patients with suspected positive lymph node status, while in contrast, Sangal et al. (2018), in a population-based study, demonstrated an improved OS in patients with advanced T-stage treated by elective neck dissection, despite cN0 status [6, 16]. Thus, it remains unclear which patients benefit from regional therapy and whether it can reduce the rate of regional recurrence [9]. Isolated regional recurrence is rare and little evidence is available demonstrating possible risk factors [1]. Beneficial factors for OS are negative surgical margins and multimodal therapy, as well as initial cN0 status [2, 17].

The aim of this study was to analyze a large patient cohort with sinonasal malignancies, focusing on the presence of regional involvement in primary diagnosis and in the case of recurrence. Long-term oncological outcome and prognostic factors for locoregional control, progression-free survival (PFS) and OS were assessed.

## Patients and methods

### Ethical considerations

The institutional and regional review board (Inselspital, Bern University Hospital, Switzerland, reference number KEK-BE 002/2015) granted approval to conduct the study. All procedures involving human participants were in accordance with the ethical standards of the national research committee and with the 1964 Helsinki declaration and its later amendments.

### Patients and data acquisition

A retrospective chart review was performed of all patients with histologically confirmed malignant tumors located in the nose and paranasal sinuses who were discussed in the multidisciplinary tumor board of the Head and Neck Anticancer Center, Inselspital, Bern University Hospital between 01/2008 and 12/2019. In order to give a complete overview on sinonasal malignant tumors, all entities were included. Collected data included patient characteristics, initial staging classification pre- and postoperatively (according to the Union for International Cancer Control TNM Classification 7th edition (2010)), treatment and follow-up. For MM, the American Joint Committee on cancer staging mucosal melanoma of the head and neck, 7th edition was applied. Locoregional MRI was used for radiological staging and, in cases of suspect lymph nodes, ultrasound with fine needle aspiration cytology of the pathological neck lymph nodes was performed. In cases of malignant cells in cytology, the treatment of the neck (surgery or RT) was performed. All treatment decisions were determined in an interdisciplinary tumor board. All patients were included in the descriptive analysis. To calculate PFS and OS, only patients with intended curative therapy were included in the statistical analysis.

### Statistical analysis

The follow‐up time was calculated starting by the date of the primary treatment and not censored at a predefined point in time. Kaplan‐Meier curves and log‐rank tests were used to depict and compare the variables regarding time‐to‐event endpoints respectively. Multivariate Cox's proportional hazards regression models with variables yielding *p* < 0.1 in univariate analyses were generated to evaluate possible prognostic factors for PFS and OS. Backward elimination was used to identify potential independent factors with a cut-off *p* < 0.05. Analyses were performed with JMP (version 14.0; SAS Institute GmbH, Heidelberg, Germany).

## Results

### Patient cohort

One hundred and forty-four patients with histologically proven sinonasal malignancies were treated in our tertiary reference center between 2008 and 2019. Median follow-up was 47 months (range 1–160). The most frequent histological type was SCC with 51%. Patient and tumor characteristics and the five most frequent histological entities are shown in Table 1. Additional rare histological entities (n = 13) were observed: sarcoma (n = 6; angiosarcoma, chondrosarcoma, leiomyosarcoma, malignant solitary fibrous tumor, primitive neuroectodermal tumor and rhabdomyosarcoma; one case each), sinonasal undifferentiated carcinoma (n = 3), adenosquamous carcinoma (n = 1), mucoepidermoid carcinoma (n = 1), non-small-cell carcinoma (n = 1) and NUT-midline carcinoma (n = 1).

**Table 1:** Patient and disease characteristics of the whole cohort and the five most frequent tumor entities

	Whole cohort (%)	Squamous cell carcinoma (%)	Adenocarcinoma (%)	Melanoma (%)	Adenoid cystic carcinoma (%)	Esthesio-neuroblastoma (%)
Total patients	144 (100)	74 (100)	24 (100)	18 (100)	9 (100)	7 (100)
Male / female	86 (59.7) / 58 (40.3)	48 (64.9) / 26 (35.1)	18 (75) / 6 (25)	6 (33.3) / 12 (66.7)	4 (44.4) / 5 (55.6)	3 (42.9) / 4 (57.1)
Median age; years (range)	61.5 (10–93)	59 (23–92)	67.8 (29–86)	74.9 (39–91)	61.1 (40–93)	63.6 (37–83)
Tumor subsite						
Nasal cavity	98 (68.1)	54 (73.0)	15 (62.5)	14 (77.8)	4 (44.4)	3 (42.9)
Paranasal sinuses	37 (25.7)	17 (23.0)	7 (29.2)	2 (11.1)	4 (44.4)	3 (42.9)
Multiple subsites	9 (6.3)	3 (4.0)	2 (8.4)	2 (11.1)	1 (11.1)	1 (14.3)
Clinical tumor classification						
T1	41 (28.5)	28 (37.9)	5 (20.1)	2 (11.1)	1 (11.1)	3 (42.9)
T2	22 (15.3)	12 (16.2)	2 (8.3)	1 (5.6)	2 (22.2)	1 (14.3)
T3	24 (16.7)	7 (9.5)	5 (20.1)	8 (44.4)	3 (33.3)	0
T4a	34 (23.6)	18 (24.3)	4 (16.7)	5 (27.8)	2 (22.2)	3 (42.9)
T4b	23 (16)	9 (12.2)	8 (33.3)	2 (11.1)	1 (11.1)	0
Clinical nodal classification						
N0	124 (86.1)	64 (86.5)	21 (87.5)	15 (83.3)	8 (88.9)	7 (100)
N1	8 (5.6)	3 (4.1)	0	3 (16.7)	0	0
N2	12 (8.3)	7 (9.5)	3 (12.5)	0	1 (11.1)	0
Clinical distant metastasis classification						
M1	7 (4.9)	3 (4)	2 (8.3)	1 (5.6)	0	0

For primary treatment, the majority of patients underwent surgery with adjuvant RT as illustrated in Table 2 for the five most frequent tumor entities and the whole cohort. Fourteen patients (10.4%) had no curative intended treatment due to the advanced stage of disease.

**Table 2:** Primary treatment of the whole cohort and the five most frequent tumor entities

		Whole cohort (%)	Squamous cell carcinoma (%)	Adenocarcinoma (%)	Melanoma (%)	Adenoid cystic carcinoma (%)	Esthesio-neuroblastoma (%)
Total patients		144 (100)	74 (100)	24 (100)	18 (100)	9 (100)	7 (100)
Primary surgery		29 (20.1)	17 (23)	5 (20.8)	1 (5.6)	1 (11.1)	2 (28.6)
Primary surgery + RT		66 (45.8)	26 (35.1)	13 (54.2)	13 (66.7)	7 (77.8)	5 (71.4)
Primary RT alone		17 (11.8)	12 (16.2)	2 (8.3)	2 (11.1)	1 (11.1)	0
Primary RTCX		18 (12.5)	14 (18.9)	1 (4.2)	0	0	0
Palliative therapy (CX ± RT)		12 (9.0)	4 (5.4)	3 (12.5)	2 (11.1)	0	0
Best supportive care		2 (1.4)	1 (0.7)	0	0	0	0
Margin status after surgery	R0	25 (27.5)	14 (35)	5 (27.8)	4 (30.8)	1 (16.7)	1 (12.5)
	R1	35 (38.5)	14 (35)	5 (27.8)	4 (30.8)	5 (83.3)	3 (37.5)
	R2	5 (5.5)	1 (2.5)	2 (11.1)	1 (7.7)	0	0
	RX	26 (28.6)	11 (27.5)	6 (33.3)	4 (30.8)	0	4 (50)

*RT* Radiotherapy, *CX* Chemotherapy

### Regional involvement and management

In the whole cohort, 20 of 144 patients (13.8%) were initially staged as cN + . These include the tumor entities shown in Table 3 and, additionally, one patient with NUT carcinoma, one sinonasal undifferentiated carcinoma and one rhabdomyosarcoma.

**Table 3:** Initial regional involvement, therapeutic management and histopathological findings of the cervical lymph nodes for the whole cohort and the five most frequent tumor entities

	Whole cohort (%)	Squamous cell carcinoma (%)	Adeno-carcinoma (%)	Melanoma (%)	Adenoid cystic carcinoma (%)	Esthesio-neuroblastoma (%)
Total patients	144 (100)	74 (100)	24 (100)	18 (100)	9 (100)	7 (100)
cN +	20 (13.8)	10 (13.5)	3 (12.5)	3 (16.7)	1 (11.1)	0
Regional RT	21 (14.6)	14 (18.9)	1 (4.2)	4 (22.2)	0	0
ND	19 (13.2)	11 (14.9)	2 (8.3)	2 (11.1)	2 (22.2)	1 (14.3)
pN0	12 (8.3)	6 (8.1)	1 (4.2)	1 (5.6)	2 (22.2)	1 (14.3)
pN1	3 (2.1)	1 (1.4)	1 (4.2)	1 (5.6)	0	0
pN2b	4 (2.8)	4 (5.4)	0	0	0	0

*ND* Neck dissection, *RT* Radiotherapy

Clinical N + status was mainly observed in locally advanced tumors. The respective T-stages of the patients were: T1 = 4, T2 = 2, T3 = 2, T4a = 2, T4b = 10.

Of the 20 patients staged cN + , ten patients received a neck dissection (± additional RT) while five patients underwentprimary chemoradiation with locoregional RT and five patients received no neck treatment due to a palliative concept. In seven of the 10 operated patients, regional involvement was confirmed by histopathology, whereas three patients were down-staged to pN0. These were: one patient with rhabdomyosarcoma, one adenocarcinoma and one adenoid cystic carcinoma. Despite a clinical status of cN0, nine patients underwent neck dissection and four patients had (loco-)regional RT according to the tumor board recommendation. Rationale for regional therapy was individual, in many cases local tumor extent (T4 stage) or required neck access for free flap reconstruction. In all cases, histopathology confirmed the pN0 status. In total, 33 patients (22.9%) underwent any elective neck treatment (Table 3).

### Recurrence and treatment

Fifty-six of 130 patients (43.1%) had a relapse after curatively intended treatment. Of these, 26 patients had local, seven loco-regional and nine isolated regional recurrences. Recurrence patterns and therapeutic management are shown in Table 4.

**Table 4:** Recurrence, its treatment and survival in the whole cohort and the five most frequent tumor entities (initially curatively treated patients only)

	Whole cohort	Squamous cell carcinoma	Adeno-carcinoma	Melanoma	Adenoid cystic carcinoma	Esthesio-neuroblastoma
Total patients	130 (100)	69 (100)	21 (100)	16 (100)	9 (100)	7 (100)
Recurrence/persistence*	56 (43.1)	23 (33.3)	12 (57.1)	9 (56.3)	5 (55.6)	3 (42.9)
Localization						
Local	26 (20)	9 (13)	8 (38.1)	4 (25)	2 (22.2)	2 (28.6)
Loco-regional	7 (5.4)	5 (7.2)	1 (4.8)	1 (6.3)	0	0
Regional	9 (6.9)	7 (10.1)	1 (4.8)	0	0	1 (14.3)
Loco-regional and distant	7 (5.4)	1 (1.4)	1 (4.8)	2 (12.5)	1 (11.1)	0
Distant only	7 (5.4)	1 (1.4)	1 (4.8)	2 (12.5)	2 (33.3)	0
Months to recurrence/persistence* or death; median (range)	10 (1°–73)	6 (1°–20)	11 (3–73)	14 (3–73)	14 (2°–33)	36 (20–71)
Neck level of regional recurrence (rcN)	I: 4 II: 7 Multiple: 2	I: 4 II: 5	II: 1 Multiple: 1	II: 1	0	Multiple: 1
Treatment						
Salvage Sx	15 (11.5)	7 (10.1)	4 (19)	2 (12.5)	1 (11.1)	1 (14.3)
Salvage Sx + RT	16 (12.3)	8 (11.6)	3 (14.2)	3 (18.8)	1 (11.1)	0
Salvage ND	13 (10)	10 (14.5)	0	1 (6.3)	0	0
Salvage RT	2 (1.5)	0	1 (4.8)	1 (6.3)	0	0
Palliation	16 (12.3)	7 (10.1)	2 (9.6)	4 (25)	1 (11.1)	1 (14.3)
Best supportive care	3 (2.3)	1 (1.4)	1 (4.8)	0	0	1 (14.3)
2nd recurrence	13 (10)	4 (5.8)	5 (23.8)	2 (12.5)	1 (11.1)	1 (14.3)
Months to death; median (range)	39 (1–160)	45 (1–147)	56 (2–128)	21 (3–160)	57 (6–150)	47 (7–112)

^*^Time point corresponding to the date of confirming biopsy or imaging

°One patient

*ND* Neck dissection, *RT* Radiotherapy, *Sx* Surgery, (total percentage)

Of the 33 patients who initially received neck treatment, 15 (45.5%) had a recurrence with the following patterns: local n = 5, loco-regional n = 3, isolated regional n = 1 and distant metastases n = 6. These included five patients who were initially staged cN0. Of the 96 patients who only had local treatment initially, 41 (42.7%) showed a recurrence with the following patterns: local n = 21, isolated regional n = 8, loco-regional n = 4 and distant metastases n = 8.

Nine patients had isolated regional recurrence: seven patients with SCC, and one with adenocarcinoma and esthesioneuroblastoma, respectively. Eight of these nine patients had no prior neck treatment due to the initial cN0 staging. One patient with SCC had an early regional recurrence little over one monthafter loco-regional surgery and received further loco-regional RT. Another patient with adenoidcystic carcinoma had local recurrence two months after resection of a T4 tumor. Four patients with SCC developed an early regional recurrence six to nine months after primary therapy after being staged cN0. Thirteen patients had salvage neck dissection and, in all cases, the regional recurrence was histopathologically confirmed.

### Oncological outcome

**Fig. 1:**
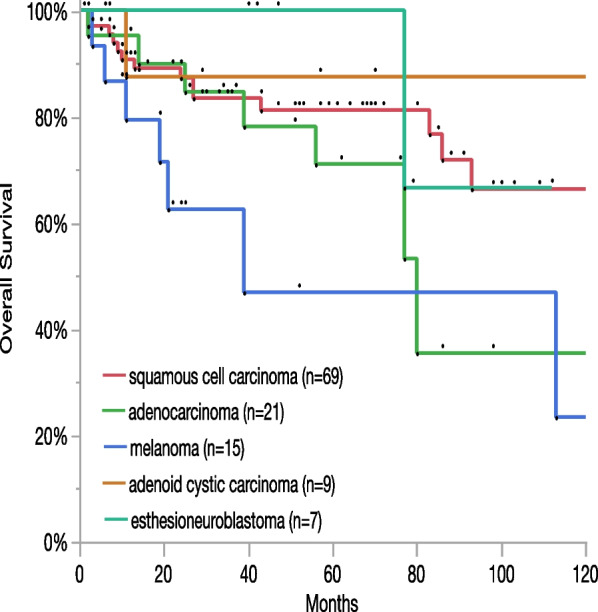
Long-term overall survival and incidence of the five most frequent entities

The oncologic outcome (initially palliatively treated cases excluded) was: no evidence of disease (n = 85, 65.4%), alive with disease (n = 8, 6.2%), dead without disease (n = 7, 5.4%), dead of disease (n = 30, 23.1%). Five-year OS of the whole cohort was 75.5%, 10-year OS was 52.1%. Progression-free survival was 47% after five and 34% after 10 years. Adenoid cystic carcinoma (87.5%) and SCC (65.1%) were entities with the highest long-term OS after ten years while MM showed the lowest with 23.5%. Kaplan–Meier curves for OS of the five most frequent entities are shown in Fig. 1.

### Multivariate analysis

For the PFS of all entities, cT > 2 (HR 2.1, 95% CI 1.3–3.6; *p* = 0.004) and cN + status (HR 2.2 (1.1–4.0); *p* = 0.034) were independent adverse risk factors in the multivariate model after backwards elimination. For OS, patient age over 60 years remained in the final model as well (HR 2.4 (1.2–5.1), *p* = 0.01; cT > 2: HR 4.4 (1.9–11.8), *p* < 0.001; cN + : HR 3.9 (1.7–8.2); *p* < 0.001). Neither the univariate nor the multivariate models yielded independent prognostic factors for locoregional control. For single entities, a multivariate model could only be established for PFS in SCC (due to low case numbers), showing solely cN + as an adverse risk factor (HR 5.6 (2.1–13); *p* < 0.001).

## Discussion

In this study, a cohort of 144 patients with sinonasal malignancies was analyzed with a focus on the clinical vs. pathological nodal status and their long-term outcome. The majority of patients (86.1%) were staged cN0 at the initial diagnosis and most patients received primary local surgery with adjuvant radiotherapy. While local recurrence was the most frequent pattern, 12.3% of the curatively treated patients had a loco-regional or isolated regional recurrence. Eight of nine patients with isolated regional recurrence had no prior neck treatment. Adenoid cystic carcinoma (87.5%) and SCC (65.1%) showed the highest long-term OS, MM (23.5%) the lowest.

Sinonasal malignancies are rare tumors with SCC being the most frequent entity also in other cohorts [1, 2, 18]. This complicates studies with large homogenous patient cohorts and prospective treatment studies. Any additional data out of large patient cohorts may therefore be helpful for further refinement of therapeutic strategies and to appropriately inform the patient prior to therapy.

For sinonasal MM it should be noted that the location in the head and neck accounts for almost half of all mucosal melanomas. This entity should be viewed separately from the other subgroups due to different treatment approaches for irresectable, metastatic or recurrent setting. Recent studies investigated the use of anti-PD1 immunooncological drugs like pembrolizumab or the combination of nivolumab and ipilimumab. In contrast, ipilimumab alone has shown less favorable results [19–21]. In the group of other tumor types, the sarcoma subgroup with six cases is too inhomogeneous to be analyzed further. Rhabdomyosarcoma, primitive neuroectodermal tumor and angiosarcoma are chemo- and radiosensitive tumors, whereas chondrosarcoma typically does respond less and solitary fibrous tumor represents a disease with clinically unpredictable behavior [22].

Few other studies present large patient cohorts of sinonasal malignancies, as most studies include only single entities (mostly SCC) and/or a single localization [14, 17, 23, 24]. In this study, SCC was the most common (51.5%) entity followed by sinonasal MM, adenocarcinoma and adenoid cystic carcinoma. This matches frequencies in most other cohorts. In analyses of national databases of the United States, SCC was the most frequent entity with 54.1% [2] and 62.4% [18] while in one European cohort adenocarcinoma was most frequent with 35.5% [13].

In non-metastatic disease, tumor resection with clean surgical margins is usually the primary goal for all entities. In this cohort, primary loco ± regional surgery with adjuvant radiotherapy was the most common curative concept. Its effectiveness in terms of OS and local control has been demonstrated for heterogeneous cohorts of sinonasal malignancies but also specifically for SCC, MM and adenocarcinoma [4–6, 10, 12, 17, 25]. However, no difference in any endpoints between primary surgery (73%) and primary radiotherapy (27%) could be demonstrated in this cohort. It is clear that therapeutic strategies have to be individual for each patient, taking into account their constitution and tumor localization, expansion and histopathology.

Regional involvement in primary diagnosis of sinonasal malignancies is rather uncommon for all entities with rates from 2 to 17% [1–3, 5, 13, 18, 26, 27]. The rate of the whole cohort (13.8%) and most frequent entities was similar. Loco-regional treatment in case of suspected regional involvement is recommended.

Less clear remains the question if any regional therapy should be administered for the majority of patients with cN0 status. There is little evidence and no clinical guideline addressing this aspect [9, 10]. In treatment studies of nasal SCC, management of the N0 neck is handled differently: Zaoui et al. [6] even performed neck dissections in only T4 patients with cN + status, which resulted in excellent OS rates above 90% in 26 patients. Hussain et al. [24] detected no occult metastases in a cohort undergoing elective neck dissections in locally advanced SCC. For SCC of the maxillary sinus, a population-based study demonstrated an improved OS in patients with T > 2 who received preventive neck dissection [16] and a meta-analysis showed a reduced risk for regional recurrence after elective neck irradiation [28]. If surgical access to the neck is required for free flap reconstruction after the resection of locally advanced tumors, a lymph node resection is commonly performed. In a 2019 review, Berger et al. concluded that no strong recommendation can be derived from literature, but elective neck dissection may be considered for locally advanced tumors as these are more likely to have occult metastases [9]. In this cohort, no occult metastases were detected and three of ten patients with cN + staging were found to be pN0. Four patients with initial cN0 staging and no neck treatment showed a regional recurrence six to nine months after primary therapy, possibly indicating a previously missed regional involvement. These observations indicate the importance of an accurate regional staging and follow-up. Isolated regional recurrence is uncommon for all entities of sinonasal malignancies and in this study occurred nearly exclusively in patients with untreated neck. This cohort showed such pattern in seven of 74 patients with SCC and in eight of 130 patients in total. In primary treatment, additional morbidity of neck treatment has to be weighed up with the possibly higher risk of regional recurrence. A structured follow-up is certainly needed to detect regional recurrences and provide the option of salvage neck treatment, which has no substantial morbidity, especially in the case of a previously untreated neck.

Local recurrence is the most frequent pattern in sinonasal malignancies while regional recurrence is less common and reported from below 10% up to 19% depending on histological type [13, 15, 29, 30]. In this cohort, the rate of regional recurrence was low (6.9%). This may be due to differences in patient cohort, follow-up and regional management. Regarding salvage therapy, curative surgery or multimodal treatment with adjuvant radiotherapy was applied in most cases. Comparing the different histopathological entities, SCC shows highest rates in recurrence and the lowest median time to recurrence in this cohort. In other cohorts, MM shows the shortest time to recurrence followed by SCC [13]. Interpretation of entities other than SCC may be imprecise because of the small number of patients in this cohort.

For long-term oncological outcome, locally advanced tumor status and nodal involvement were adverse prognostic factors in this cohort. Though these findings might be compromised because of different biological behavior of tumor types, these were also factors associated with worse OS in a large population-based cohort along multimodal therapy, histology (MM) and location in paranasal sinuses [2]. Agarwal et al. showed that socioeconomic and insurance status may also have a negative impact on OS and on treatment decision [18]. Histology was no predictive factor in multivariate analyses, but SCC and adenoid cystic carcinoma showed the best long-term OS.

Given the rare incidence of sinonasal malignancies, a prospective registry could be helpful, as well as the collaboration among hospitals with experience in these types of tumors. In the European Reference Network EURACAN for rare solid cancers, family “G7” is devoted to cancers of the head and neck with a subdomain for nasal cancer [3, 31]. Thus, the development of clinical practice guidelines could be made possible in order to fill the need for evidence and to clarify recommendations for treatment of the neck lymph nodes.

A potential strength of the study is the high number of patients and the long follow-up over a timespan of more than 10 years. The main limitations of this study are its retrospective design and the heterogeneity of entities. During the long timespan of the study, clinical practice and guidelines have changed. It is therefore challenging to form definitive conclusions out of a single-center patient cohort.

## Conclusions

This study gives a comprehensive overview on sinonasal malignancies including a broad variety of histopathological and clinical entities. Regional involvement and recurrence are uncommon. A structured follow-up of the cervical lymph nodes is critical to detecting regional recurrences, especially in patients with previously untreated necks. As there is little evidence on structured therapeutic management of the neck, centralized treatment and collaboration could be helpful to establish clinical practice guidelines.

## Data Availability

All data will be made available by the corresponding author upon reasonable request.

## Abbreviations

SCC: Squamous cell carcinoma
MM: Mucosal melanoma
OS: Overall survival
RT: Radiotherapy
PFS: Progression-free survival
